# Secondary Structure in Enzyme‐Inspired Polymer Catalysts Impacts Water Oxidation Efficiency

**DOI:** 10.1002/advs.202402234

**Published:** 2024-04-17

**Authors:** Graziela C. Sedenho, Steffane Q. Nascimento, Marjon Zamani, Frank N. Crespilho, Ariel L. Furst

**Affiliations:** ^1^ São Carlos Institute of Chemistry University of São Paulo (USP) São Carlos SP 13566‐590 Brazil; ^2^ Department of Chemical Engineering Massachusetts Institute of Technology Cambridge MA 02139 USA; ^3^ Department of Chemistry Federal University of São Carlos (UFSCar) São Carlos SP 13565‐905 Brazil

**Keywords:** water oxidation, bioinspired catalyst, multicopper oxidases, poly‐histidine, copper complex

## Abstract

Protein structure plays an essential role on their stability, functionality, and catalytic activity. In this work, the interplay between the *β*‐sheet structure and its catalytic implications to the design of enzyme‐inspired materials is investigated. Here, inspiration is drawn from the active sites and *β*‐sheet rich structure of the highly efficient multicopper oxidase (MCO) to engineer a bio‐inspired electrocatalyst for water oxidation utilizing the abundant metal, copper. Copper ions are coordinated to poly‐histidine (polyCuHis), as they are in MCO active sites. The resultant polyCuHis material effectively promotes water oxidation with low overpotentials (0.15 V) in alkaline systems. This activity is due to the 3D structure of the poly‐histidine backbone. By increasing the prevalence of *β*‐sheet structure and decreasing the random coil nature of the polyCuHis secondary structures, this study is able to modulates the electrocatalytic activity of this material is modulated, shifting it toward water oxidation. These results highlight the crucial role of the local environment at catalytic sites for efficient, energy‐relevant transformations. Moreover, this work highlights the importance of conformational structure in the design of scaffolds for high‐performance electrocatalysts.

## Graphical abstract

Conformational changes mimicking protein secondary structural changes impact the catalytic activity of the water oxidation reaction using a bio‐inspired poly‐histidine/Cu(II) catalyst.

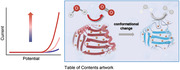

## 1 Introduction

Enzymes have evolved over billions of years to enable highly efficient, specific reactions to occur at ambient temperatures and pressures. Critical to this activity is the local environment of protein active site, with secondary‐sphere interactions facilitating favorable energetics for substrate conversion.^[^ ^1^, ^2^ ^]^ Though many important conversions can be enzymatically driven, reactions relevant to sustainability are of high interest, including carbon dioxide capture and conversion,^[^ ^3^, ^4^ ^]^ nitrogen fixation,^[^ ^5^, ^6^ ^]^ and water splitting.^[^ ^7^, ^8^, ^9^ ^]^ Despite the advantages of enzymes, their application in clean energy technologies has remained limited due to inherent challenges in the production and stability of these proteins. Many enzymes rely on metal centers for catalytic activity, which can be difficult to recombinantly express without additional machinery, and many metalloenzymes are unstable outside of the native cell environment. These challenges with expression and instability further translate to limited utility in scaled technologies.

Because of these challenges with native systems, significant effort has been devoted to the development of bio‐inspired materials that recapitulate the activity of enzymes in materials that are more stable and scalable.^[^ ^10^ ^]^ However, especially for polymeric systems, bio‐inspired materials remain limited in their control over the geometry of chelated metals serving as active sites and often have even less control over mimicking the secondary sphere influences that occur in proteins. Thus, the development of bio‐inspired materials that afford the specificity and selectivity of enzymes while maintaining the stability and scalability of polymers is essential to reach yields relevant for work beyond the laboratory.

For clean energy conversions, multicopper oxidases (MCOs) serve as an essential model family of proteins. This group is diverse and is broadly characterized by their activity, which involves the oxidation of a substrate and reduction of dioxygen.^[^ ^11^, ^12^ ^]^ The catalytic activity of this enzyme family is attributed to the three‐dimensional copper coordination sites within the protein structure.^[^ ^1^, ^8^, ^13^, ^14^ ^]^ Further, secondary structure is critical for the function of MCOs, specifically the presence of a particular orientation of *β*‐sheets. These *β*‐sheet arrangements afford MCOs the capacity to interact precisely with substrates, making the formation of similar structures in bio‐inspired materials critical.

One specific multicopper oxidase (MCO), bilirubin oxidase (BOD), is of particular interest for water splitting because it is capable of electrocatalyzing the reduction of O_2_ to H_2_O at small overpotentials at near‐neutral pH,^[^ ^11^, ^12^ ^]^ and can also catalyze to water oxidation.^[^ ^8^, ^9^ ^]^ This set of reactions represents a fundamental underpinning for renewable energy processes and has been harnessed to develop technologies including electrochemical water splitting for green hydrogen production.^[^ ^7^, ^8^, ^9^ ^]^ The inherent structural rigidity afforded by the *β*‐sheets in BOD both shields this protein during catalysis, endowing it with the resilience necessary to be effective, while also ensuring optimal interactions with substrates.^[^ ^13^ ^]^ However, this enzyme still suffers from critical challenges with stability and long‐term activity, preventing its use in many cases and requiring significant system engineering when it is implemented.

Here, we report a bio‐inspired heterogeneous electrocatalyst derived from electropolymerized histidine that forms *β*‐sheet‐like structures. To date, much of the work on bio‐inspired catalysis has focused on homogeneous catalysts, but the advantages of heterogeneous catalysis necessitates equivalent studies. The ability to consistently generate bio‐inspired heterogeneous electrocatalysts affords the advantages of homogeneous catalysts while maintaining active sites adjacent to the electrode.

Yet, techniques used for conventional heterogeneous catalyst characterization are often ineffective for soft materials such as bio‐inspired polymers. Additionally, the techniques used for solution‐phase characterization of biomolecules are not translatable to surfaces. Thus, challenges remain with establishing the local chemical environments in these materials. Techniques that have provided insights into metal chelation sites and local secondary structure include X‐ray photoelectron spectroscopic (XPS) and Fourier transform infrared (FTIR), which we use here to determine the chemistry of copper binding as well as the presence of protein‐like secondary structure in our polymers.

**Figure 1:**
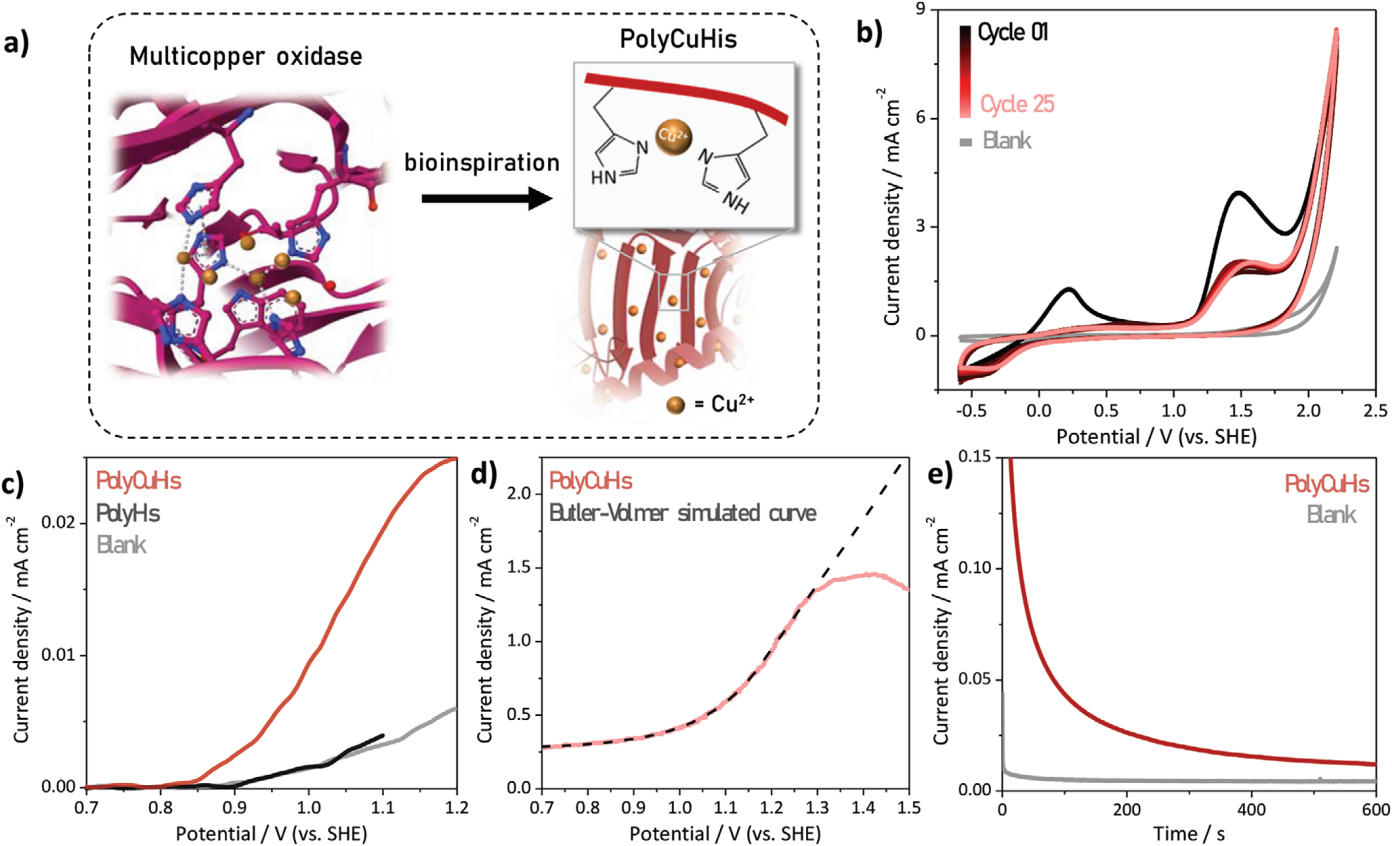
Histidine electropolymerization for multi‐copper oxidase (MCO)‐like bio‐inspired materials. a) Catalytic site of an MCO (PDB ID: 2XLL) and our polyCuHis inspired by this structure. b) Electropolymerization of polyCuHis on glassy carbon (GC) by cyclic voltammetry at 100 mV s^−1^ in 0.1 m sodium phosphate solution (pH 9.0) containing 20 mm Cu(His)_2_. c) Voltametric curves at a scan rate of 2 mV s^−1^ after subtraction of the capacitive current for polyCuHis, polyHis, and bare GC (blank) in 0.1 m sodium phosphate solution (pH 9.0). (d) Voltametric curve at 50 mV s^−1^ recorded with polyCuHis in 0.1 m sodium phosphate solution (pH 9.0) and the Butler–Volmer simulated curve. e) Chronoamperometry at 1.21 V recorded with polyCuHis and bare GC (blank).

Electropolymerized histidine is formed directly on electrodes, serving as a spatially‐controlled scaffold to chelate metal ions as catalysts active sites at the electrode surface. We incorporated copper ions into the poly‐histidine scaffold, forming poly‐copper histidine (polyCuHis). The copper (II) ions are coordinated to histidine residues on a polypeptide chain, akin to the active sites of MCOs (**Figure** **1**a), while supporting heterogeneous electrocatalysis. This material shows water oxidation catalysis when secondary structures resembling *β*‐sheets are present. Decreased catalyst efficiency is observed when the material loses the characteristics of these protein secondary structures. Thus, we can use this material to determine the role of *β*‐sheet structure in its catalytic activity. These findings confirm the critical importance of secondary sphere interactions driven by secondary structure in bio‐inspired catalytic materials, and our combined characterization strategies enable elucidation of these interactions in our heterogeneous catalysts.

## 2 Results and Discussion

### 2.1 Materials Design and Synthesis

Based on the structure of MCOs, in which the copper ion in the active site is chelated by histidines, we selected histidine as a monomer for bio‐inspired polymer generation. Further, the ability of histidine to be electropolymerized enables the generation of polyhistidine under mild aqueous conditions. We can additionally incorporate copper ions into this matrix using a prepared Cu(His)_2_ complex to directly generate polyCuHis electrochemically on a glassy carbon (GC) surface (Figure S1, Supporting Information). To generate a sufficient polymer layer at the electrode surface, cyclic voltammetry over 25 successive cycles was performed in a 0.1 m sodium phosphate solution (pH 9.0) containing 20 mm Cu(His)_2_ (Figure 1b). In the initial voltammetric cycle, two oxidation peaks at 0.22 and 1.48 V are observed, attributed to the Cu(His)_2_ oxidation. The decrease in the oxidative peak currents and the positive potential shift in faradaic currents in subsequent cycles indicate histidine polymerization on the electrode surface.^[^ ^15^ ^]^ Electropolymerization was confirmed through scanning electron microscopy (SEM) and spectroscopic analyses (see Section S2, Supporting Information). These methods further confirmed the consistent deposition of the polyCuHis polymer. The simplicity and aqueous compatibility of the electropolymerization reaction negates the need for complex synthetic pathways and enables the reproducible generation of these materials.

Because the chelated copper ion is essential for catalytic activity, we next confirmed that copper remains in the polyhistidine matrix post‐polymerization. The copper interactions with the polymer were evaluated by XPS and FTIR (Figures S2–S4, Supporting Information). From these data, an atomic ratio of 0.23 for Cu/N was determined. This ratio suggests that one copper ion is coordinated for every one to two histidine residues in the polymer matrix. This ratio is consistent with the expected stoichiometry from the Cu(His)_2_ precursor. The maintenance of this ratio is indicative of stable Cu‐His chelation throughout the polymerization process. Further, these results indicate that the copper is chelated to the nitrogen atoms of imidazole and the amines of the poly‐histidine backbone, as well as to water molecules. This coordination pattern is expected for a copper‐histidine complex prepared at pH 9. These data confirm the formation of a copper‐containing histidine polymer in which the metal active site is chelated to the imidazole groups as it is in native MCOs. Together, these data demonstrate the generation of an electropolymerized material with structural characteristics similar to native proteins.

### 2.2 Catalytic Control

As the catalytic centers were confirmed to be incorporated in the polyCuHis, the (bio)electrocatalytic abilities of this material were then evaluated under similar conditions to those previously employed for BOD‐catalyzed water oxidation.^[^ ^8^ ^]^ Quasi‐steady‐state currents (Figure 1c) indicate that the onset potential for the water oxidation reaction (WOR) is 0.85 V, corresponding to an overpotential of 0.15 V for the O_2_/H_2_O reaction (*E* ^0^ = 0.70 V vs SHE, pH 9.0). In contrast, no significant oxidative currents are observed with the bare GC electrode (without polyCuHis) or with the histidine polypeptide in the absence of coordinated Cu^2+^ ions (Figure S6, Supporting Information). The overpotential observed for this polymer is comparable to MCO and is smaller than those reported for copper‐based catalyst complexes.^[^ ^16^, ^17^, ^18^ ^]^ Additionally, this overpotential is lower than that of the soluble Cu(His)_2_ complex (Figure S5, Supporting Information). These results confirm that the polyCuHis can behave as a water oxidation catalyst with equivalent efficiency to enzymes and with higher efficiency than conventional small‐molecule catalysts.

In contrast, no catalysis was observed on bare glassy carbon electrodes or on electrodes with polyhistidine but no copper. These results confirm that the electrocatalytic WOR behavior is due to the copper ions coordinated within the histidine polypeptide. At 50 mV s^−1^ (Figure 1d), oxidative faradaic currents are observed at potentials higher than 0.9 V, reaching a maximum current density of 1.46 mA cm^−2^ at 1.40 V. The WOR overpotential to reach 1.00 mA cm^−2^ is 0.51 V, which is smaller than those previously reported for other copper‐based complexes (Table S1, Supporting Information). When the amount of (bio)electrocatalyst on the electrode surface was decreased by performing only ten CV cycles for electropolymerization, the polyCuHis on the electrode produced a smaller maximum current density (1.05 mA cm^−2^ at 1.31 V, Figure S7, Supporting Information), as expected. The catalytic behavior deviates from the Butler–Volmer predicted kinetic behavior (Equation S1, Supporting Information), which predicts that current will have an exponential dependence on the applied potential. As the reactant is in large excess in our system, mass‐transfer effects are not the cause of the kinetic deviation. Hence, the deviation from the Butler–Volmer behavior can be attributed to decreased kinetics at the electrode at high potentials.

Chronoamperometric measurements are consistent with the cyclic voltammetry. At 1.21 V (an overpotential of 0.51 V, Figure 1e), the bare glassy carbon electrode has minimal background oxidative current (4 µA cm^−2^), while the polyCuHis reaches a steady‐state current of 13 ± 2 µA cm^−2^. Further, the water oxidation current was found to be related to the total amount of polyCuHis electropolymerized on the electrode surface, with higher currents at electrodes with more polyCuHis. Similarly, when polyCuHis was electropolymerized on carbon cloth electrodes, which have significantly larger electrochemical surface areas than their geometric areas (Figure S8, Supporting Information), the steady‐state catalytic current density reaches 0.10 mA cm^−2^ (Figure S9, Supporting Information). These results show that the current density is not only dependent on the polyCuHis but also on the electrochemical surface area of the carbon‐based material, as is expected for a process dependent on direct interaction with the electrode.

To determine the efficiency of the WOR, the O_2_ produced from WOR by polyCuHis during the constant potential measurements at 1.21 V was monitored. The O_2_ was electrochemically detected by a Pt‐based O_2_ detection system, in which increasingly negative current is measured from the reduction of the O_2_ generated (Figure S10, Supporting Information). Further, the absence of undesired side products commonly generated during water oxidation was confirmed. Colorimetric detection of the main possible reaction byproduct (H_2_O_2_) using an enzymatic assay showed no discernable peroxide generation; following 600 s of electrolysis at 1.21 V (Figure S11, Supporting Information), no H_2_O_2_ was detected (Figure S12, Supporting Information). These results confirm that the observed electrocatalysis generated the desired O_2_ product.

Interestingly, following chronoamperometric measurements, we noted a rapid decline in current density. Yet neither copper nor biopolymer was detected in the electrolyte (Figures S13 and S14, Supporting Information). As nothing was found dissociated from the electrode, these results suggest that a conformational change surrounding the active sites i responsible for the observed current decrease. We hypothesized that the applied potential could cause a conformational change in the polyCuHis, ultimately inhibiting catalysis. Conformational changes leading to catalytic inhibition further explain the deviation from Butler–Volmer behavior (Figure 1d). Similar inhibition has been reported for MCOs, which is attributed to transport limitations of the necessary substrates, especially at sub‐optimal pH values.^[^ ^7^, ^8^, ^9^ ^]^

**Figure 2:**
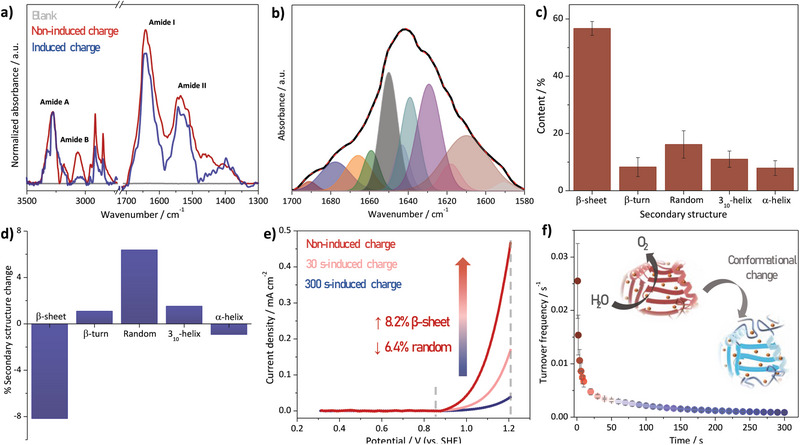
a) FTIR spectra of native polyCuHis, after charge induction, and the bare electrode. b) Zoomed view of amide I band (black line) of no induced charge polyCuHis, the deconvoluted curves (filled curves), and their sum (dashed red line). c) Content of each secondary structure in polyCuHis, obtained from deconvoluted amide I band in (b). d) Changes in the secondary structure contents of polyCuHis with charge induction. e) Forward voltammograms at 50 mV s^−1^ after subtraction of the capacitive currents with freshly prepared polyCuHis and after charge induction at 1.21 V for 30 and 300 s. f) Turnover frequency values with the charge induction at 1.21 V, and representation of polyCuHis conformational change.

We therefore investigated the effects of applied potential on the conformation of polyCuHis using micro‐Fourier transform infrared (FTIR) spectroscopy (**Figure** **2**a). Compared to other techniques, such as circular dichroism, which are often limited in their ability to provide detailed information about protein structure and dynamics due to the inherent effects of light scattering, particularly in the UV–vis region, FTIR spectroscopy does not suffer from these limitations and enables studies in the solid‐state rather than solution, better recapitulating the local environment of the polyCuHis on electrodes. Characteristic polypeptide absorption bands are observed in the spectra of both the induced and non‐induced charge (in the presence or absence of an applied potential) for polyCuHis films, such as amide A (3280 cm^−1^), amide B (3067 cm^−1^), amide I (1643 cm^−1^), and amide II (1533 cm^−1^) (Table S2, Supporting Information). We obtained and analyzed 384 spectra at different regions of the polyCuHis film. As the dipole moments of the amide I and II are approximately perpendicular, the change in the intensity ratio of these two bands from 1.65 ± 0.20 (non‐induced charge) to 2.60 ± 0.04 (induced charge) suggests conformational changes in the polypeptide adsorbed on the electrode surface.

The amide I band provided further insight into the potential conformational changes occurring in the polymer matrix (Figure 2b; Figure S15, Supporting Information). Upon deconvolution of this band in the non‐induced charge sample, polyCuHis was found to be primarily composed of *β*‐sheet (56.7 ± 2.4%), a result expected for a polyHis backbone synthesized in alkaline media (Figure 2c).^[^ ^19^ ^]^ Additionally, smaller amounts of *β*‐turn (8.3 ± 3.3%), random coil (16.1 ± 4.7%), 3_10_ helix (11.0 ± 2.8%), and *α*‐helix (7.9 ± 2.6%) were identified. The *β*‐sheet conformation is more extended than helices, forming zigzag polypeptide chains that can be arranged side by side to create a structure resembling a series of pleats in which copper ions are coordinated. The 3_10_ helix, on the other hand, is an intermediate in α‐helix formation, and *β*‐turn and random coil regions can connect structured *β*‐sheet and helix regions. After charge induction, differences in the amide I band of polyCuHis were observed (Figure S16, Supporting Information), indicating conformational changes. The decrease of 8.2% in the *β*‐sheet content and the increase in 6.4% of random structure content (Figure 2d) suggest that the applied potential can induce charges in the histidine residues, resulting in changes to the 3D polypeptide conformation. However, no significant variations in the overall content of *β*‐turn and helices were found.^[^ ^20^ ^]^

These findings suggest that the conformational changes in polyCuHis are complex and not simply attributable to changes in secondary structure. Notably, it appears that longer periods of applied potential have a more pronounced effect on the catalytic activity (Figure 2e). The combination of electrochemical and spectroscopic measurements suggests that a more extensive and disordered structure is formed under these conditions due to charge repulsion by the potential application. Although the conformational variation of polyCuHis is not extensive, electrochemical results indicate that this variation significantly impacts electrocatalytic activity toward WOR, highlighting the crucial role of the 3D arrangement of catalytic sites within the polypeptide backbone for effective reactivity. This is evidenced by a decay in turnover frequency (number of water molecules oxidized per Cu^2+^ site per second) with the induction charge at 1.21 V (Figure 2f, see calculation in the Supporting Information). The initial turnover frequency at 1 s of reaction is 0.26 s^−1^ and decreases exponentially over the first 50 s, with a decay observed until 300 s.

Though both our polyCuHis material and native MCO proteins show similar current decreases in response to lengthy WOR processes, we attribute the decreased activity in our bio‐inspired system to conformational changes rather than substrate kinetic limitations or direct inhibition. MCOs are known to have the active copper‐histidine sites protected and stabilized within the protein shell, whereas in the polyCuHis, the histidine residues coordinated to copper ions can experience significant changes in their local chemical environment due to the impact of the applied potential. The applied potential induces charges in the polypeptide chain, which can lead to conformational changes in the polyCuHis that alter catalysis.

Taken together, our results demonstrate the critical role of local environment on catalysis in bio‐inspired systems. Specifically, here, we generate polyhistidine chelated with copper ions to mimic BOD‐like MCOs. Highly efficient water oxidation catalysis was observed with these materials without generating harmful peroxide by‐products. Interestingly, a decay in the current generated from these catalysts is observed and is dependent on the length of time a potential is applied. We find changes in secondary structure, specifically percentage of the polymer that forms *β*‐sheets, to change upon application of a potential, highlighting the importance of the three‐dimensional environment of catalytic sites in synthetic catalysts, as well as the impact of small conformational changes that enzymes can experience due to the environmental conditions. Our findings provide critical design guidelines for the generation of scalable, inexpensive bio‐inspired catalysts for clean energy‐relevant transformations.

## Conflict of Interest

The authors declare no conflict of interest.

## Supporting information

Supporting Information

## Data Availability

The data that support the findings of this study are available in the supplementary material of this article.
