# Supplementary material for: Secondary Structure in Enzyme‐Inspired Polymer Catalysts Impacts Water Oxidation Efficiency
Source: Adv Sci (Weinh). 2024 Apr 17;11(25):2402234. doi: 10.1002/advs.202402234 (PMC11220705; doi:10.1002/advs.202402234)
Supplement: Supplementary file 1 — Supporting Information [file ADVS-11-2402234-s001.pdf]

ADVANCED SCIENCE
Open Access

Supporting Information

for *Adv. Sci.*, DOI 10.1002/advs.202402234

Secondary Structure in Enzyme-Inspired Polymer Catalysts Impacts Water Oxidation Efficiency

Graziela C. Sedenho, Steffane Q. Nascimento, Marjon Zamani, Frank N. Crespilho and Ariel L. Furst**

SUPPORTING INFORMATION

Secondary Structure in Enzyme-Inspired Polymer Catalysts Impacts Water Oxidation Efficiency

Graziela C. Sedenho[1,2]†, Steffane Q. Nascimento[1], Marjon Zamani,[2] Frank N. Crespilho[1]*, and Ariel L. Furst[2]*

[1] *São Carlos Institute of Chemistry, University of São Paulo (USP), São Carlos, SP 13566-590, Brazil.*
[2] *Department of Chemical Engineering, Massachusetts Institute of Technology, Cambridge, MA 02139, United States.*
† *Current address: Department of Chemistry, Federal University of São Carlos, São Carlos, SP 13565-905, Brazil.*

*Corresponding authors: frankcrespilho@iqsc.usp.br; afurst@mit.edu

Contents

S1. Experimental 2
S1.1. Reagents and materials 2
S1.2. Electrochemical measurements 2
S1.3. Copper-histidine complex synthesis and electropolymerization 2
S1.4. Micro-FTIR spectroscopy 3
S1.5. Scanning electron microscopy (SEM) and energy dispersive X-ray spectroscopy (EDS) 3
S1.6. X-ray photoelectron spectroscopy (XPS) 4
S1.6. Inductively coupled plasma mass spectrometry (ICP-MS) 4
S2. Morphological and spectroscopic characterizations of PolyCuHis. 5
S3. Copper-histidine complex for water oxidation 9
S4. Electropolymerization of polyHis 10
S5. Comparison of WOR catalytic current 11
S6. Varying the total polyCuHis amount 12
S7. Butler-Volmer model 12
S8. Electrode surface area and polyCuHis on CC electrode 13
S9. Electrochemical detection of the produced O_2 15
S10. Enzymatic colorimetric H_2O_2 detection 16
S11. Determination of copper ion release following the application of an electrochemical potential 18
S12. Optical images of polyCuHis, FTIR signal assignments and evaluation of amide I band ... 19
S13. Determining turnover frequency (TOF) 21
References 22

S1. Experimental

S1.1. Reagents and materials

Sodium phosphate monobasic (NaH_2PO_4, 141.96 g mol^{-1}, ≥99.0%), sodium hydroxide pellets (NaOH, 40.0 g mol^{-1}, ≥98%), L-histidine ($C_6H_9N_3O_2$, 155.15 g mol^{-1}, ≥99%), copper sulfate pentahydrate ($CuSO_2.5H_2O$, 249.69 g mol^{-1}, 99.995%), and isopropanol (C_3H_8O, 60.1 g mol^{-1}, >99%) were purchased from Sigma-Aldrich (US). All aqueous solutions were prepared with ultrapure water generated from an ELGA PURELAB Quest UV (Model number: PQDIUVM1NSP). The pH of phosphate solution was adjusted to 9.0 with 5 M NaOH solution. Glassy carbon (GC, geometric area = 0.07 cm^2) from CH Instruments (CHI104), carbon cloth (CC, geometric area = 1.0 cm^2) from Fuel Cell (Panex 30 Fabric PW06), and highly ordered pyrolytic graphite (HOPG, geometric area = 1.44 cm^2) from Bruker were employed as working electrodes.

S1.2. Electrochemical measurements

Electrochemical measurements were performed using a Gamry 600 Reference potentiostat (Gamry Instruments, Warminster, PA, USA), using Pt wire and an Ag/AgCl electrode (3 M NaCl filling solution) as counter and reference electrodes respectively, and glassy carbon (GC), carbon cloth (CC), or highly oriented pyrolytic graphite (HOPG) as working electrode. The potential values were converted to values against the standard hydrogen electrode (SHE) using the formula $E_{\mathrm{SHE}} = E_{\mathrm{Ag/AgCl}} + 0.21$, where E_{SHE} is the potential value in Volts versus SHE and $E_{\mathrm{Ag/AgCl}}$ is the measured potential value in Volts versus the Ag/AgCl electrode. All potential values are reported against SHE. The measured currents were converted to current densities by normalizing to the geometric area of the working electrode. All measurements were performed at ambient temperature.

Prior to electrochemical measurement, GC was cleaned by polishing with a 0.05 μm alumina slurry, followed by sonication in isopropanol and deionized water for 5 minutes. CC was sonicated in 70% (v/v) ethanol and water for 10 min. HOPG was cleaned using the scotch tape method. A piece of tape was pressed onto the HOPG surface and then pulled off to remove the top layers of the HOPG. The edge-plane surfaces were isolated with nitrocellulose resin, to expose only the HOPG clean basal-plane surface to the electrolyte.

S1.3. Copper-histidine complex synthesis and electropolymerization

Copper-histidine complex $Cu(His)_2$ was prepared by mixing Cu^{2+} and histidine at the 1:2 molar ratio. For that, 20 μL of 1.0 M $CuSO_4$ solution in ultrapure water was added to 200 μL of 0.2 M L-histidine solution previously prepared in 0.1 M sodium phosphate solution (pH 9.0). The final concentration of $Cu(His)_2$ was 91.0 mM. The color of $CuSO_4$ solution changes from light to dark blue when added to L-histidine solution, indicating complexation. The featured band at 640 nm in the UV-visible spectrum solution confirms the $Cu(His)_2$ formation (Figure S1).[19] For electropolymerization on the electrode surface, a suitable volume of freshly-prepared $Cu(His)_2$ complex solution was added to 0.1 M sodium phosphate solution (pH 9.0) to a final concentration

of 20.0 mM. A potential was then applied between -1.01 to 2.21 V at 100 mV s^{-1} for 25 cycles to induce polymerization, as previously described.[20] Subsequently, the electrode was rinsed three times with ultrafiltered water.

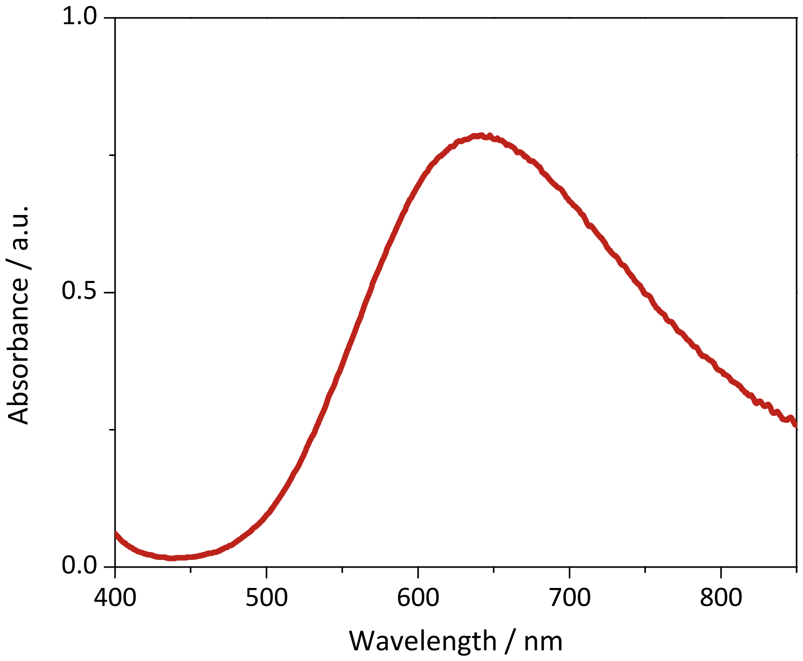

Figure S1. UV-visible spectrum of 10 mM $Cu(His)_2$ in 0.1 M sodium phosphate solution (pH 9.0).

S1.4. Micro-FTIR spectroscopy

Spectroscopic measurements in the infrared region (IR) were performed in a Vertex 70v FTIR spectrometer (Bruker, Germany). The spectra were collected in the reflectance mode between 4,000 and 1,300 cm^{-1} at 64 accumulations with a 4 cm^{-1} resolution in six different regions of each sample. Optical images were collected with a 36× objective lens. For the micro-FTIR spectroscopic measurements, polyCuHis was electropolymerized on an HOPG surface. The samples were rinsed three times with ultrafiltered water and dried under vacuum for at least 30 min prior to analysis.

S1.5. Scanning electron microscopy (SEM) and energy dispersive X-ray spectroscopy (EDS)

The measurements were recorded using a Zeiss Merlin High-resolution scanning electron microscope (Jena, Germany) with an accelerating voltage of 16 kV. PolyCuHis was electropolymerized on a CC surface as described in Section S1.3. The samples were rinsed in ultrapure water and dried under vacuum overnight before the measurements. Samples were attached on a typical pin stub using carbon conducting tape and silver paste prior to the analyses.

S1.6. X-ray photoelectron spectroscopy (XPS)

XPS measurements were performed at Brazilian Nanotechnology National Laboratory (LNNano), part of the Brazilian Centre for Research in Energy and Materials (CNPEM). A Thermo Scientific K-Alpha X-ray photoelectron spectrometer with a monochromatic Al Kα X-ray (hν = 1486 eV) excitation source was used. All spectra were measured using a spot size of 300 μm and 10 scans. The XPS survey spectrum was recorded at pass energy of 200.0 eV, energy step size of 1.00 eV, and dwell time of 10 ms. The high-resolution spectra at C 1s, N 1s, O 1s, and Cu 2p regions were recorded at pass energy of 50.0 eV, energy step size of 0.10 eV, and dwell time of 50 ms. PolyCuHis was electropolymerized on carbon cloth and rinsed three times with ultrafiltered water and dried under vacuum for at least 30 min prior to analysis.

S1.6. Inductively coupled plasma mass spectrometry (ICP-MS)

An Agilent 7900 IC-PMS (serial # JP15180835) was used to measure copper (^{63}Cu). The samples were measured with helium gas, and germanium (^{72}Ge) was used as an internal standard. Test and calibration samples (3.0 mL) in 0.1 M sodium phosphate solution (pH 9.0) were lyophilized and then resuspended and incubated in 1.5 mL 60% HNO_3 overnight. Next, they were diluted in ultrapure water with 2% HNO_3 and filtered through a 0.22 μm filter prior analyses.

S2. Morphological and spectroscopic characterization of PolyCuHis.

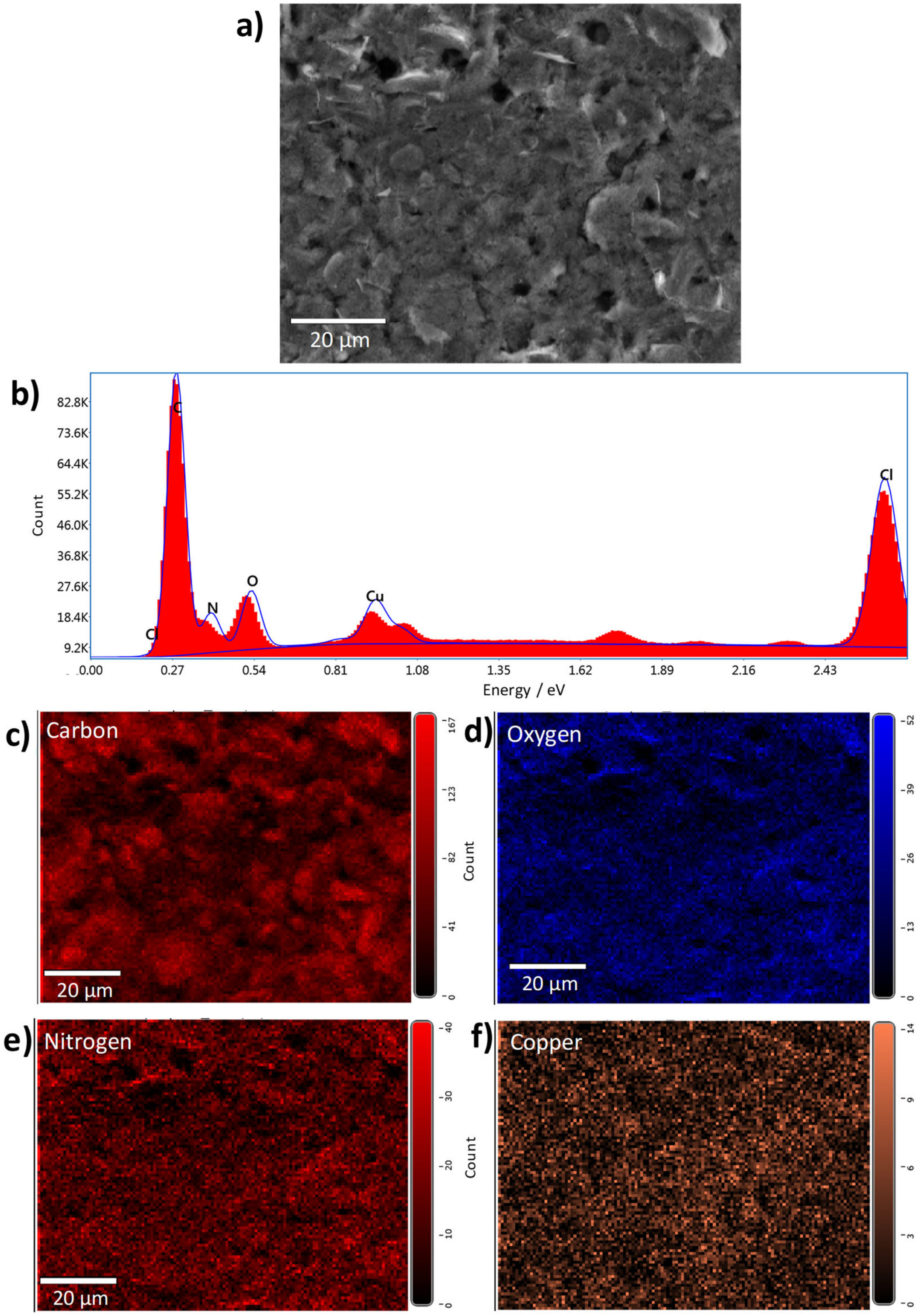

Figure S2. (a) SEM image of polyCuHis film and (b) its EDX spectrum indicating the presence of carbon, oxygen, nitrogen copper and chlorine. (c) Carbon, (d) oxygen, (e) nitrogen, and (f) copper map in image (a).

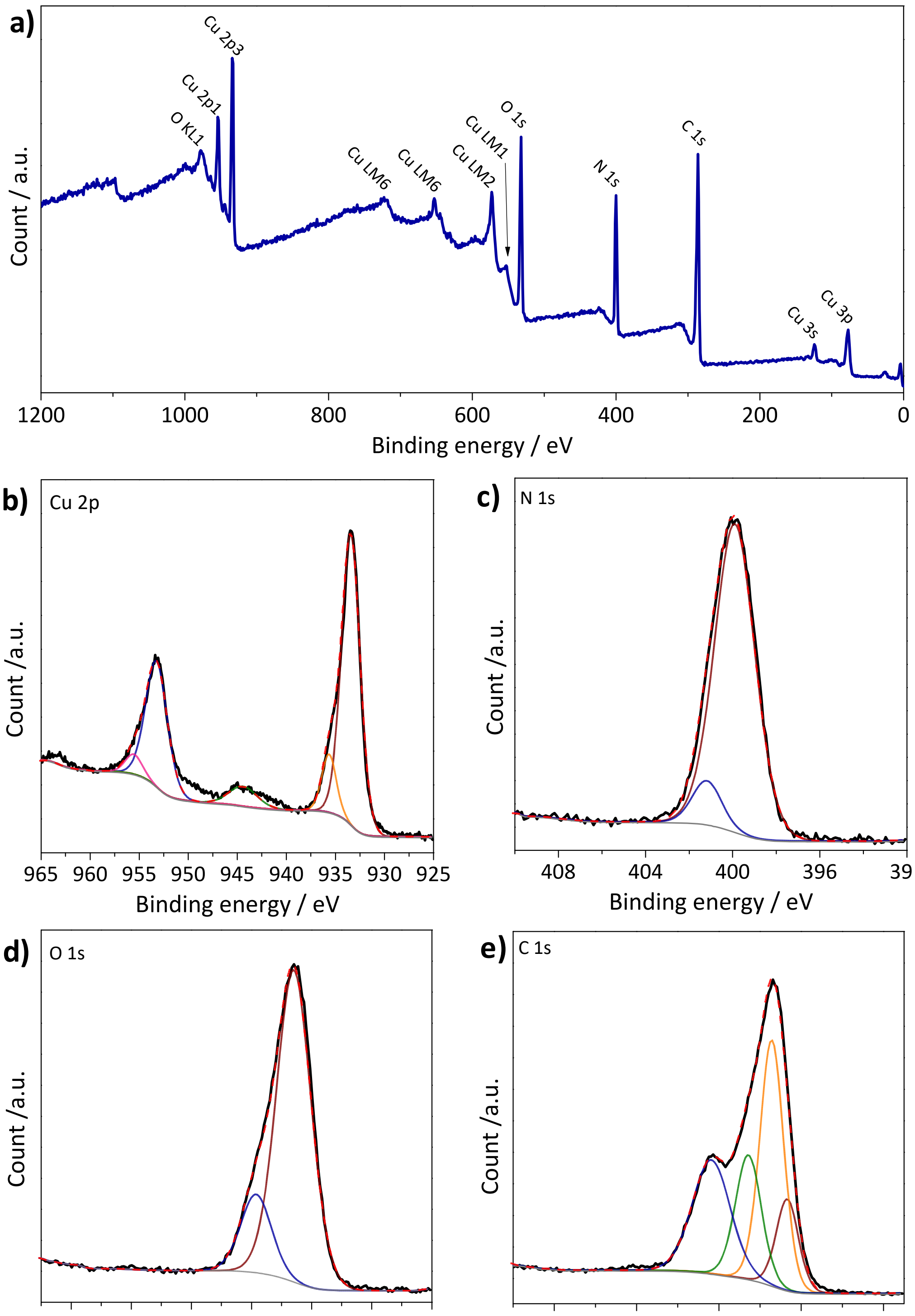

Figure S3. (a) XPS survey spectrum of polyCuHis film. High resolution spectra in (b) Cu 2p, (c) N 1s, (d) O 1s, and (e) C 1s regions, where solid black lines represent raw spectra, wine, blue, green, orange, and pink solid lines are the deconvoluted peaks and dashed red lines are the sums of the deconvoluted peaks.

XPS survey spectrum (Figure S3a) shows the presence of Cu, N, O, and C atoms, expected to polyCuHis electropolymerized on carbon cloth. Cu/N atomic ratio was calculated being 0.23, which suggests that one copper ion is coordinated for 1-2 histidine residues. These insights into the coordination environment enable optimization of the scaffold to enhance electrocatalysis.

High-resolution spectrum in Cu 2p region shows the copper characteristic peaks at 933.4 and 953.2 eV (Figure S3b), which are features of Cu(II) $2p_{3/2}$ and Cu(II) $2p_{1/2}$. The satellite peak at 944 eV indicated that the chelation of copper in the form of Cu(II).[21,22] Cu(I) compounds in contrast to paramagnetic Cu(II) compounds do not show satellites in Cu 2p spectra.[22] The spectrum profile is very different from that expected for copper oxides and copper hydroxide,[23] indicating that those species are not formed on the electropolymerization process. The deconvolution of high-resolution spectrum in N 1s region (Figure S3c) displays two peaks, at 401.2 eV, which are assigned to Cu–N,[24] and at 399.9 eV, attributed to amide (N–C=O), amine ($C–NR_2$, where R is C and/or H), and C-N=C groups.[24,25] This result evidences the presence of Cu coordinated for histidine by N atoms. The deconvoluted high-resolution spectrum in O 1s region (Figure S3d) also shows two peaks are assigned to carbonyl (C=O, 531.9 eV) and carboxylic (–COOH, 533.7 eV) groups. The deconvolution of the high-resolution spectrum in O 1s region (Figure S3e) evidences four peaks at: 284.6 eV, associated to C-C and C=C; 285.4 eV, attributed to $C–NH_2$; 286.5 eV assigned to C=O, C–OH, C–O–C, O=C–NH; and 288.3 eV, corresponding to –COOH.[25–27] The functional groups observed in the oxygen and carbon high-resolution spectra can be attributed to histidine and/or groups on the carbon fibers where the polyCuHis was electropolymerized.

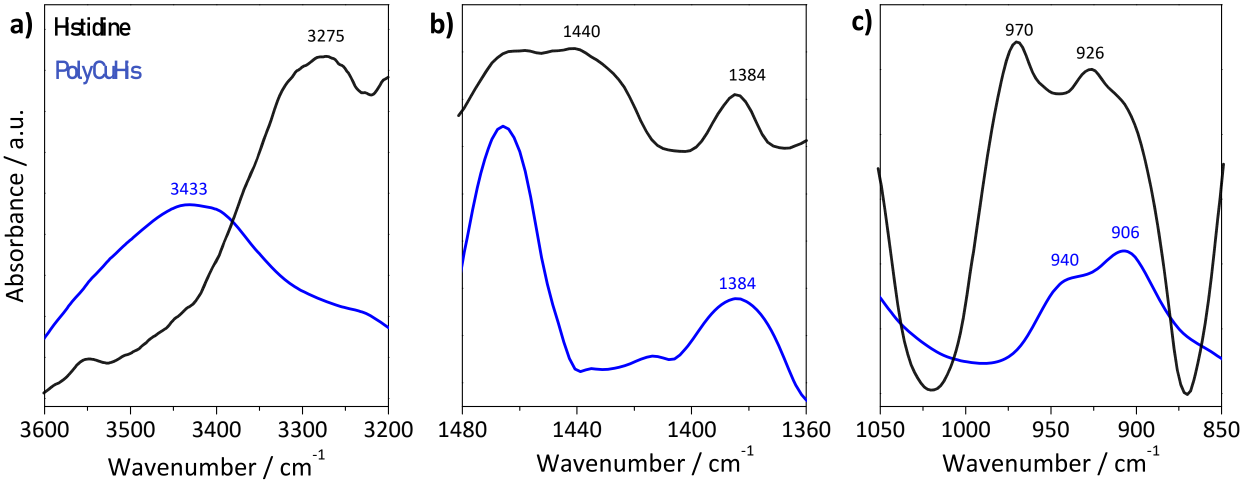

Figure S4. Zoomed views of the FTIR spectra of polyCuHis (blue curves) and standard histidine (black curves) in (a) high, (b) middle, and (c)small wavenumber regions.

For a further investigation about the copper coordination to histidine in polyCuHis, FTIR measurements were performed and compared to standard histidine (Figure S4). The involvement of the amine group and water molecules in coordination can be supported by the analysis of the

higher wavenumber region of the spectrum (Figure S4a), although the band broadening due to H-bonds made the assignments difficult. The band at 3267 cm^{-1} in the histidine spectrum, attributed to the symmetric streaching of NH_2 group,[28] is suppressed in the polyCuHis spectrum, possibly indicating the formation of amidic bound and coordination with Cu(II). The appearance of a band at 3433 cm^{-1} in polyCuHis spectrum (Figure S4b) is assigned to O–H stretching, and suggests the coordination of H_2O molecules to Cu(II).[28] In addition, the band at 1440 cm^{-1} in the histidine spectrum, featured of and CN stretching and NH(ring) and CH_2 in-plane bending modes,[10,11] are suppressed in the polyCuHis spectrum, indicating the coordination of Cu(II) to the N atoms from histidine imidazole group. The signal at 1384 cm^{-1}, attributed to the COO^- symmetric stretching,[30,31] do not change in histidine and polyCuHis spectra, revealing that the histidine carboxylic groups are not involved in the copper ion coordination. The copper ion coordination to N atoms is also supported by changes in the vibrational modes at smaller wavenumbers (Figure S4c). The signals at 970 cm^{-1}, assigned to NH(ring) out of plane bending, CN stretching, CH_x and imidazole in plane bending modes,[28,29] and at 926 cm^{-1}, corresponding to N3C4 stretching,[32] shift to smaller wavenumbers (940 and 906 cm^{-1}, respectively), indicating coordination to the copper ion.

S3. Copper-histidine complex for water oxidation

The small molecule copper-histidine complex ($Cu(His)_2$) was investigated as a homogeneous electrocatalyst for the WOR. Cyclic voltammograms were recorded with GC in 0.1 M sodium phosphate solution (pH 9.0) containing 10.0 mM $Cu(His)_2$ (Figure S5). The onset potential for the WOR oxidation is 0.95 V.

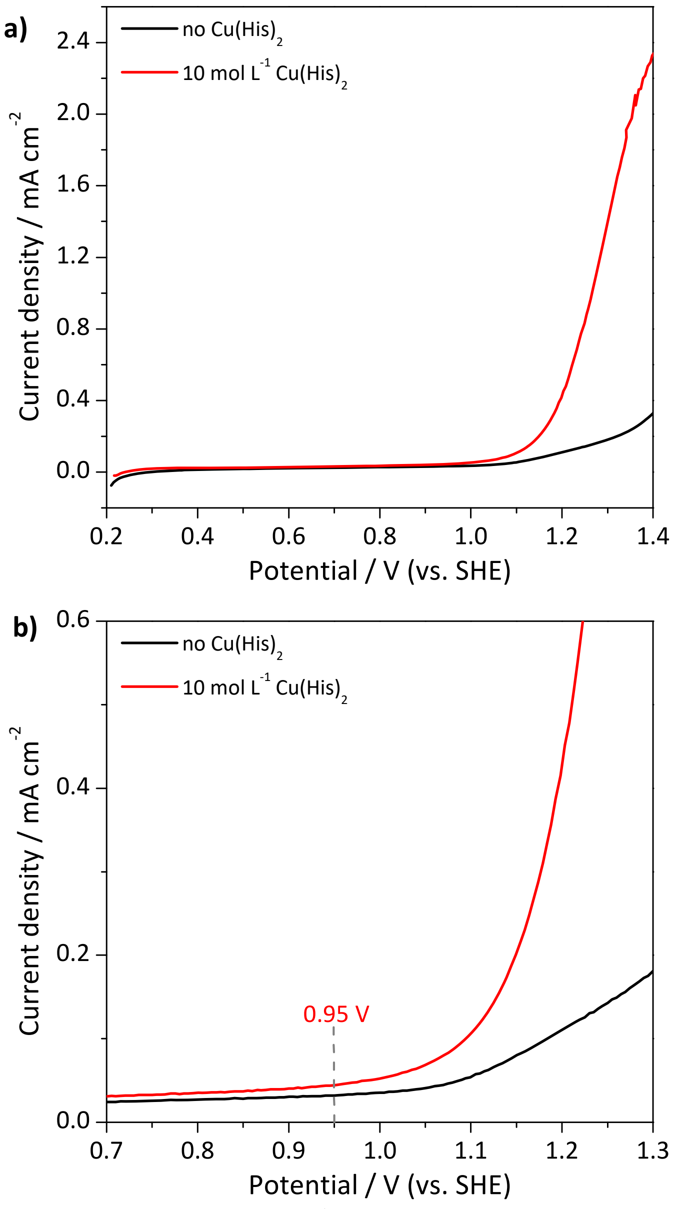

Figure S5. (a) Voltammograms at 50 mV s^{-1} recorded with GC electrode in 0.1 M sodium phosphate electrolyte solution (pH 9.0). Scans were obtained in the absence (grey) and presence (red) of 10 mM $Cu(His)_2$. (b) Zoomed view of the voltammogram showing the onset potential for the WOR.

S4. Electropolymerization of polyHis

PolyHis in the absence of coordinated Cu^{2+} was electropolymerized on GC surfaces through the application of 25 successive potential cycles between -1.01 and 2.21 V at 100 mV s^{-1} in 0.1 M sodium phosphate solution (pH 9.0) containing 20.0 mM His monomer (Figure S6). The oxidation peak at 1.48 V in the initial voltametric curve is related to the histidine monomer oxidation. The decrease in the oxidation peak currents and the peak potential shift to more positive values over the successive potential cycles indicate the histidine polymerization on the electrode surface.[20,33]

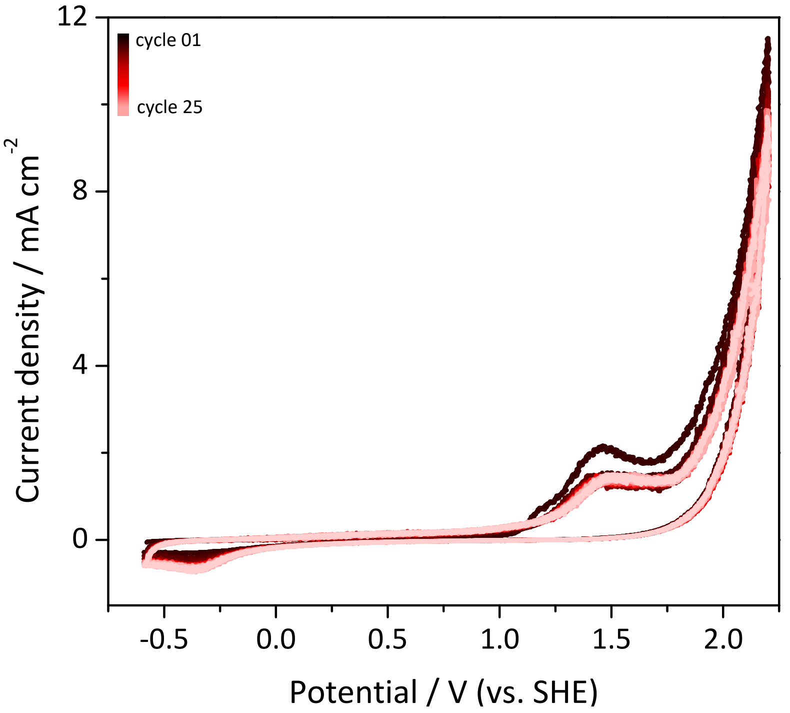

Figure S6. (a) Electropolymerization of polyHis on GC by cyclic voltammetry at 100 mV s^{-1} in 0.1 mol L^{-1} sodium phosphate solution (pH 9.0) containing 20 mM His.

S5. Comparison of WOR catalytic current

The WOR overpotential to reach 1.00 mA cm^{-2} obtained with polyCuHis was compared to those previously reported for other copper-based complexes. The overpotential values and the conditions that they were obtained are summarized in Table S1. The thermodynamic potential (E0) for the WOR was calculated by equation S1:

Table S1. Electrochemical performance of reported copper-based complexes for WOR catalysis.

Cu(II)-based catalyst	Method	Experimental potential at 1.0 mA cm^{-2}	E^0 vs SHE at the experimental pH	Calculated overpotential	Ref.
polyCuHis	Voltammetry at 50 mV s^{-1}, pH 9.0	1.21 V	0.70 V	0.51 V	This work
$Cu(TCA)_2$	Voltammetry at 100 mV s^{-1}, pH 6.0	≈ 1.9 V	0.88 V	1.02 V	[34]
$[(DAM)Cu_3(\mu^3\text{-}O)][Cl]_4$ **	Voltammetry at 100 mV s^{-1}, pH 8.1	≈ 1.3 V	0.75 V	0.55 V	[35]
	Voltammetry at 30 mV s^{-1}, pH 6.0	> 1.6 V		> 0.85 V	
$Cu(pyalk)_2$ ***	Chronoamperometry at 1.1 V, pH 12.5	1.1 V	0.49 V	0.51 V	[36]

* TCA = 1-Mesityl-1H-1,2,3-triazole-4-carboxylic acid

** DAM = dodecaaza macrotetracycle

*** pyalk = 2-pyridyl-2-propanoate.

S6. Varying the total polyCuHis amount

PolyCuHis was electropolymerized on GC surfaces by 10 successive potential cycles between -1.01 and 2.21 V at 100 mV s^{-1} in 0.1 M sodium phosphate solution (pH 9.0) containing 20.0 mM $Cu(His)_2$. The electrode was subsequently rinsed three times with ultrapure water, followed by transfer to fresh 0.1 M sodium phosphate solution (pH 9.0) for WOR evaluation (Figure S7).

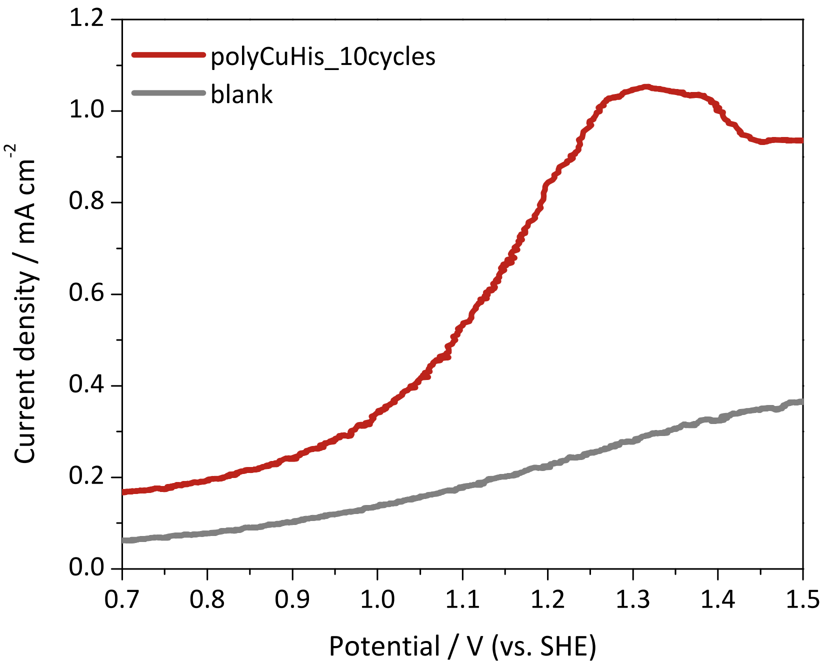

Figure S7. Cyclic voltammograms at 50 mV s^{-1} recorded with polyCuHis formed by 10 cycles of electropolymerization (red) and bare GC (blank, grey) in 0.1 M sodium phosphate solution (pH 9.0).

S7. Butler-Volmer model

The Butler-Volmer equation (Equation S1) determines the current at a specific overpotential (E-$E^{o'}$) in the absence of mass-transfer impacts or limitations.

$$i = i_0 \left[\mathrm{e}^{-\alpha \frac{\mathrm{F}}{RT}(E-E^{0'})} - \mathrm{e}^{(1-\alpha)\frac{\mathrm{F}}{RT}(E-E^{0'})} \right] \quad \text{(S1)}$$

where, i is current, i_0 is the exchange current (balanced faradaic anodic or cathodic current at equilibrium), α is the transfer coefficient, F and R are the Faraday and gas constants, respectively, E is the applied potential and $E^{0'}$ the formal potential. For The Butler-Volmer curve was simulated to fit the experimental voltametric data at low potentials (0.70-1.25 V).

S8. Electrode surface area and polyCuHis on CC electrode

The electrochemical surface area of the carbon electrodes was determined by cyclic voltammetry. Quantitatively, larger capacitive current (capacitive current = anodic current – cathodic current) indicates higher electrical double layer capacitance of the electrode and is a reliable indicator of electrochemical surface area. Representative cyclic voltammograms of GC and CC obtained in 0.1 M sodium phosphate electrolyte solution (pH 9.0) in a non-faradic potential region are displayed in Figure S8. The capacitive current densities of GC and CC at 0.40 V are 0.10 ± 0.01 and 0.37 ± 0.02 mA cm^{-2}, respectively. This indicates that the electrochemical surface area of CC is higher than that of GC by a factor of 3.7.

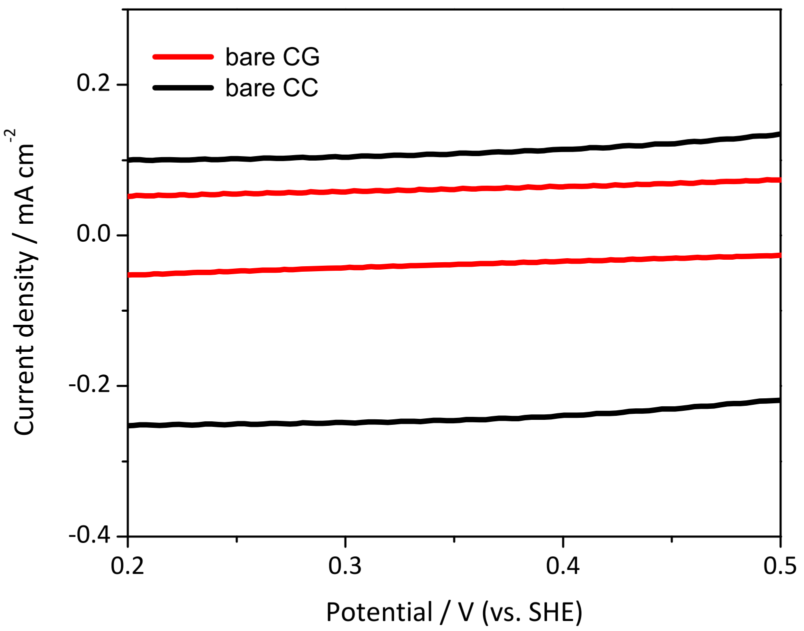

Figure S8. Cyclic voltammograms of CC and GC at 100 mV s^{-1} in 0.1 M sodium phosphate solution (pH 9.0).

PolyCuHis was electropolymerized on CC electrode as described in Section S1.3. Constant potential measurements at 1.21 V (Figure S9) show the steady-state current for WOR with this polymer to be 0.105 mA cm^{-2} (red), whereas the bare CC (blank, grey) shows an oxidative current of 0.044 mA cm^{-2}.

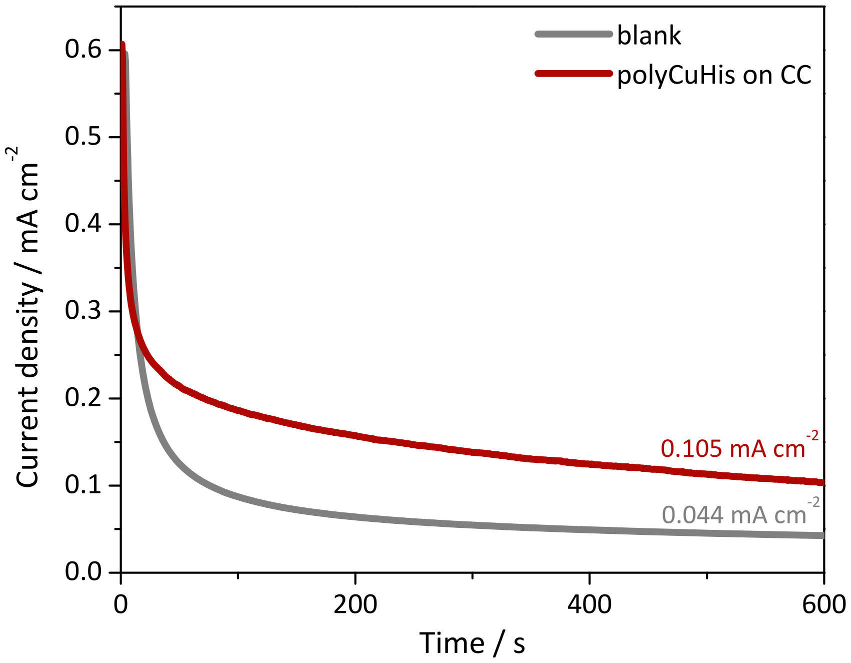

Figure S9. Chronoamperometry curve at 1.21 V recorded with polyCuHis on CC (red) and bare CC (blank, grey).

S9. Electrochemical detection of the produced O_2

To detect the generated O_2 from the WOR catalyzed by polyCuHis, classical amperometric sensor based on the oxygen reduction reaction on Pt was used.[37,38] The O_2 detection is based on the O_2 reduction on a Pt wire polarized at -0.30 V (vs SHE), by current monitoring with time. The sensor was coupled to the electrochemical cell where water is catalytically oxidized by polyCuHis polarized at 1.21 V (same potential used in the TON calculation) for in situ O2 detection. The system was calibrated in the presence and absence of O2 dissolved in the electrolyte (phosphate solution, pH 9.0). The response curve (blue curve in Figure S10) shows the electroreduction of the produced O_2 a few seconds after 1.21 V is applied to the polyCuHis-modified CC (at 100 s, indicated by ON in Figure S10). The increase in the current density to more negative values (from 0.05 mA cm^{-2} at 100 s to -0.23 mA cm^{-2} at 690 s) indicates the electroreduction of the O_2 produced by the polyCuHis. As soon as the WOR potential application was interrupted (indicated by OFF in Figure S10), the measured O_2 reduction current decreases. A control amperometric curve was recorded applying 1.21 V (vs SHE) at an unmodified carbon cloth electrode (Figure S10, grey curve).

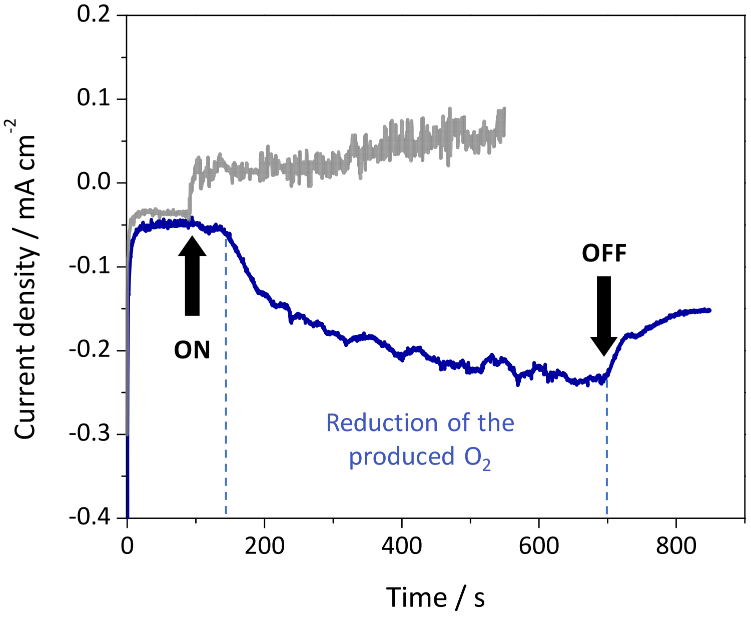

Figure S10. Chronoamperometry curves recorded with the Pt-based O_2 sensor while 1.21 V is applied to polyCuHis on CC (blue) and bare CC (grey).

S10. Enzymatic colorimetric H_2O_2 detection

As H_2O_2 is not the desired WOR product but can be produced as an electrolysis by-product, we investigated whether this chemical was produced in our system. H_2O_2 quantification was performed in triplicate using horseradish peroxidase (HRP) reaction-driven 2,2'-azinobis(3-ethylbenzothiazoline-6-sulfonic acid) (ABTS) color development (see equation S2).

$$H_2O_2 + \text{ABTS} \xrightarrow{\text{HRP}} 2\ H_2O + \text{oxidized ABTS} \quad \text{(S2)}$$

PolyCuHis electropolymerized on a CC electrode (0.7 cm^2) was employed for water electrolysis at 1.21 V for 600 s in 2.5 mL of 0.1 M sodium phosphate solution (pH 9.0), as shown in Figure S11. The oxidation charge was calculated by the integration of each curve to be 0.110, 0.112, and 0.091 C for electrodes #1, #2, and #3, respectively. The molar concentration of H_2O_2 produced considering 100% of current efficiency as calculated by equation S3 to be 1.35 ± 0.12 mM.

$$c_{H_2O_2} = \frac{Q}{n\text{F}V} \quad \text{(S3)}$$

where, $c_{H_2O_2}$ is the H_2O_2 concentration (mol L^{-1}), Q is the charge (C) obtained from the integration of the current-time curves, n is de number of electrons involved in the reaction (2 electrons, in this case), F is Faraday constant (9,485 C mol^{-1}), and V is reaction volume (0.0025 L, in this case).

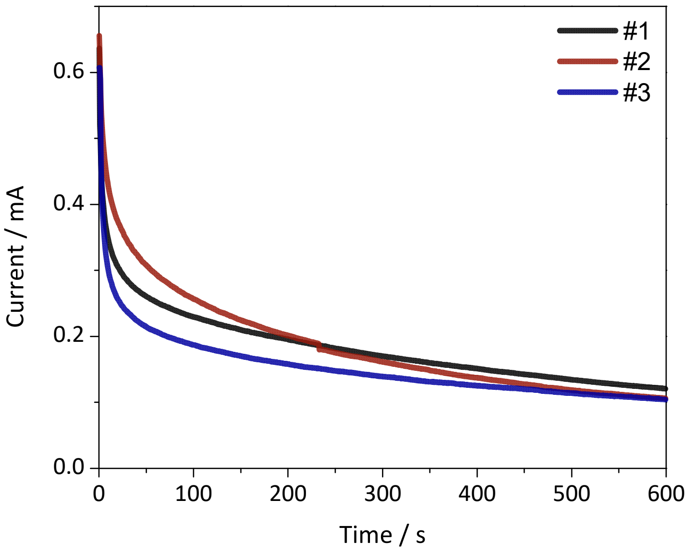

Figure S11. Chronoamperometry curves at 1.21 V recorded with CC electrodes modified with polyCuHis.

The effective H_2O_2 concentration produced by electrolysis can be determined by enzymatic assay with HRP enzyme and ABTS (equation S3). For that, a calibration curve with standard H_2O_2 (2-75 μM H_2O_2) was built (Figure S12a). The linear fit generated an equation $y = 0.1208 + 0.0224x$, with R^2 equals to 0.9996.

After the electrolysis, the electrolyte pH was adjusted to 5.0 by addition of suitable volume (approximately 20 μL) of 37% HCl. In a 96-well plate, 188 μL of each sample was added to a freshly prepared 2 μL of 0.05 μg mL^{-1} HRP (Thermo Scientific, prod. no. 21134) and 2 μL of 20 mM ABTS (final concentration = 1 mM) in 0.1 M phosphate buffer (pH 5.0). After 15 min of reaction, the absorbance at 410 nm of three electrolyte samples was measured at 25 °C, as well as the absorbance of a blank solution (1 mM ABTS in 0.1 M phosphate buffer, pH 5.0), as control (Figure S12b). No considerable absorbance change was observed compared to the control solutions, indicating that no significant H_2O_2 was found in the electrolyte samples.

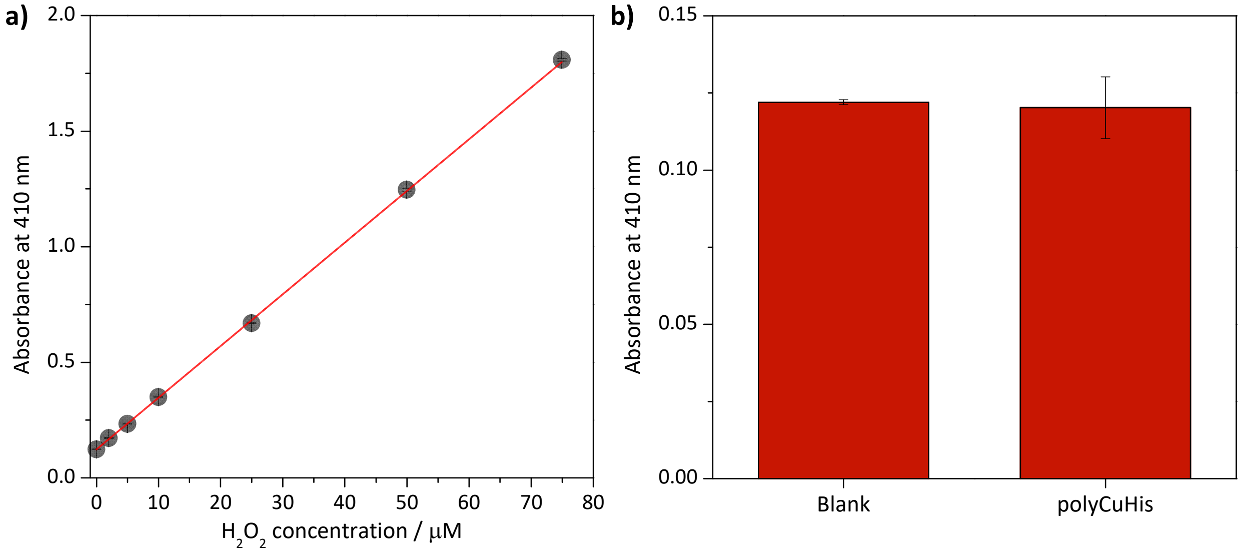

Figure S12. (a) H_2O_2 calibration curve. (b) Absorbance value at 410 nm of blank solution (1 mM ABTS in 0.1 M phosphate buffer, pH 5.0) and the electrolyte after electrolysis with polyCuHis on CC electrode.

S11. Determination of copper ion release following the application of an electrochemical potential

To determine whether copper ions are released from the polymer upon an applied potential for WOR, ICP-MS was used. A copper calibration curve was generated, and the electrolyte was collected following application of 1.21 V for 600 s at the polyCuHis-modified electrode (Figure S13b, samples #1-3). A blank sample was prepared in fresh electrolyte as a control. Samples were prepared as described in Section S1.6. Additionally, a freshly polymerized electrode was incubated in 60% HNO_3 overnight to dissolve the polyCuHis and the solution was analyzed for total Cu determination on the electrode (Figure S9b, sample #4). Copper quantification was accomplished through the generation of a calibration curve (Figure S13a). The linear fit generated an equation $y = 7979.5x + 4.855 \times 10^5$, with $R^2 = 0.998$. Copper was then quantified following subtraction of the blank scan from each sample. Concentration was then corrected to incorporate subtraction of the blank from each sample following correction for the dilution factor employed in the sample preparation. Concentration was then corrected to incorporate dilution factor. Only negligible copper was found in the electrolyte (samples #1-3) following the application of an electrochemical potential, suggesting that copper is not released from the modified electrode. The total Cu content on the electrode (sample #4) was calculated to be 1.24×10^{-7} mol cm^{-2}.

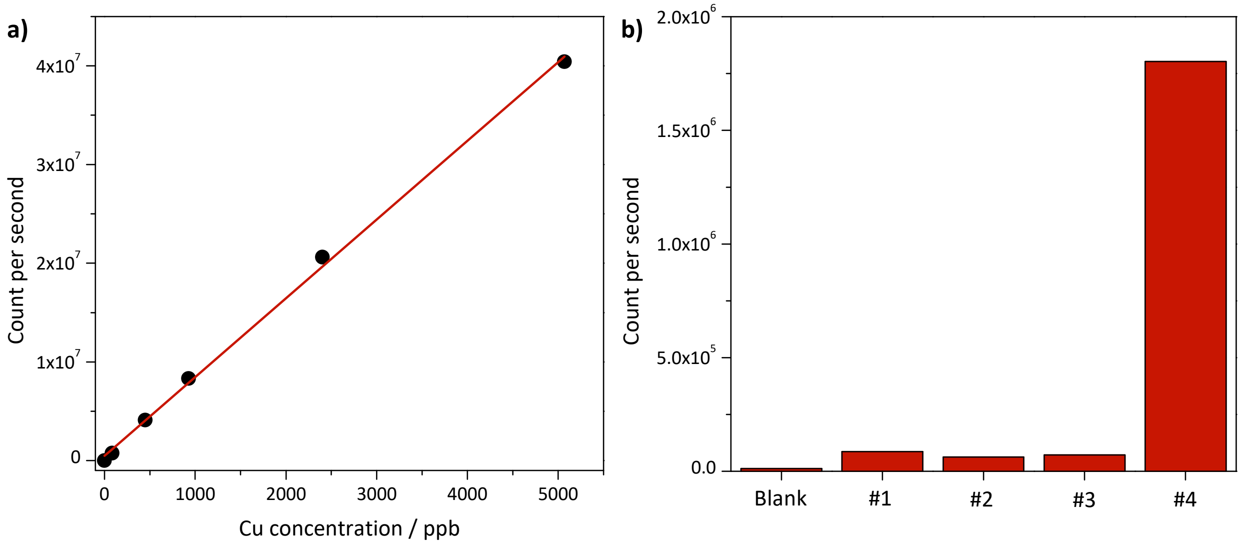

Figure S13. (a) Copper calibration curve by ICP-MS and (b) copper signal measured in each sample and in the blank solution.

S12. Optical images of polyCuHis, FTIR signal assignments and evaluation of amide I band

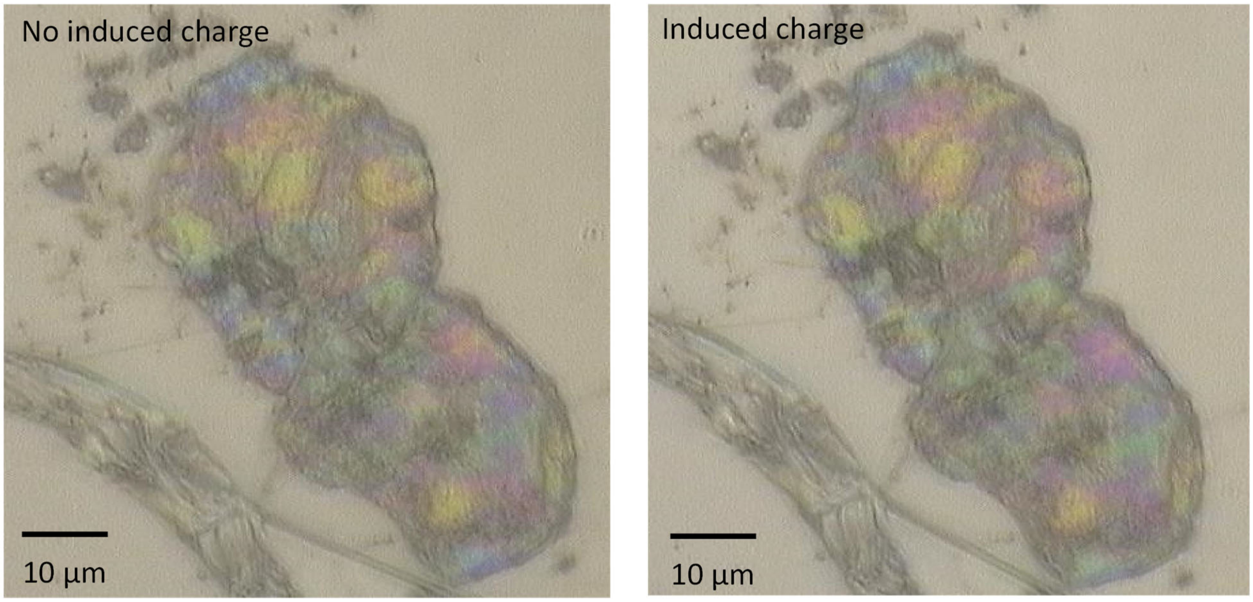

Figure S14. Optical images of polyCuHis electropolymerized on HOPG electrode before (non-induced charge, left) and after application of 1.21 V (induced charge, right).

Table S2. Assignments for polyCuHis FTIR signals.

Wavenumber / cm^{-1}	Attribution	Ref.
3280	Amide A (ν(NH))	[39]
3067	Amide B (ν(NH))	[39]
2915-2850	ν_s/ν_{as} (CH_2)	[40]
1643	Amide I (ν(C=O))	[40]
1533	Amide II (ν(CN) and ν(CH))	[39]
1458	His, $\delta_{as}(CH_3)$, $\delta(CH_2)$, ν(CN)	[40]
1394	$\nu_s(COO^-)$	[40]

ν: stretching; ν_s: symmetric stretching; ν_{as}: asymmetric stretching; δ: bending; δ_{as}: asymmetric bending.

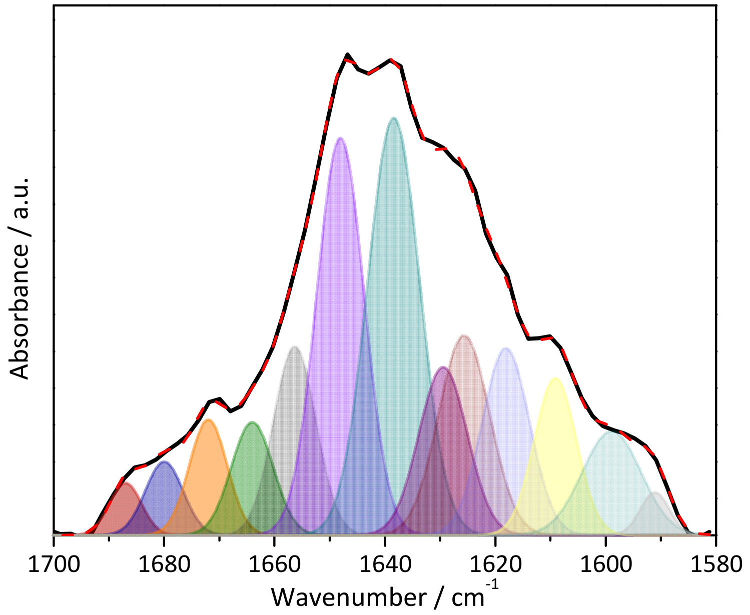

Figure S15. Zoomed view of amide I band (black line) of charge-induced polyCuHis, the deconvoluted curves (filled curves), and their sum (dashed red line).

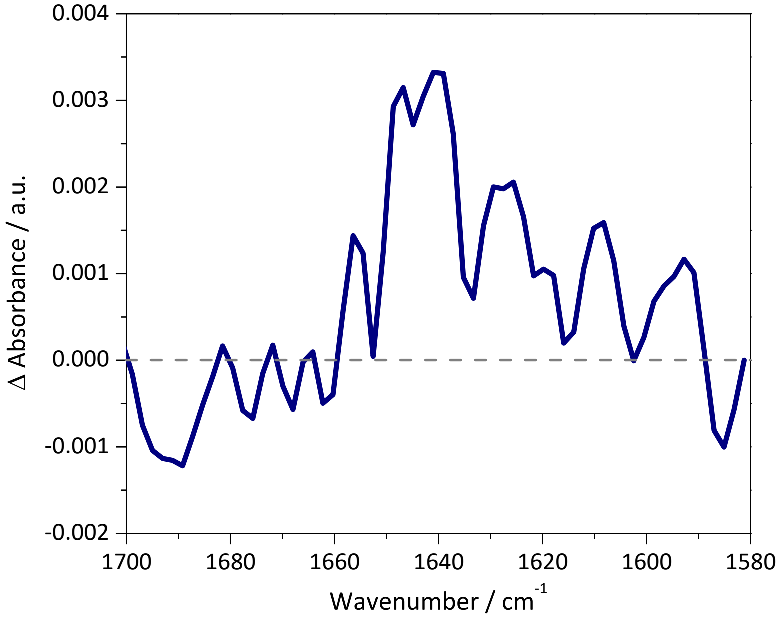

Figure S16. Changes in the amide I band of polyCuHis with the charge induction.

S13. Determining turnover frequency (TOF)

The surface excess (Γ) of copper sites on the electrode surface was calculated according to Equations S4 based on the copper anodic peak charge (Equation S5 and Figure S17) in the first cycle of electropolymerization.

$$\Gamma = \frac{Q}{n\,\mathrm{F}\,A} \quad \text{(S4)}$$

$$Q = \int \frac{i}{v}\,\mathrm{d}E \quad \text{(S5)}$$

where, Q is the charge of anodic peak (C), n is the number of electrons, F is Faraday constant (96,485 C mol^{-1}) and A is the geometric area of the electrode (cm^2), i is the faradaic current, v is the scan rate (V s^{-1}), and E is the applied potential (V). Γ of copper was determined to be 5.95×10^{-5} mol cm^2.

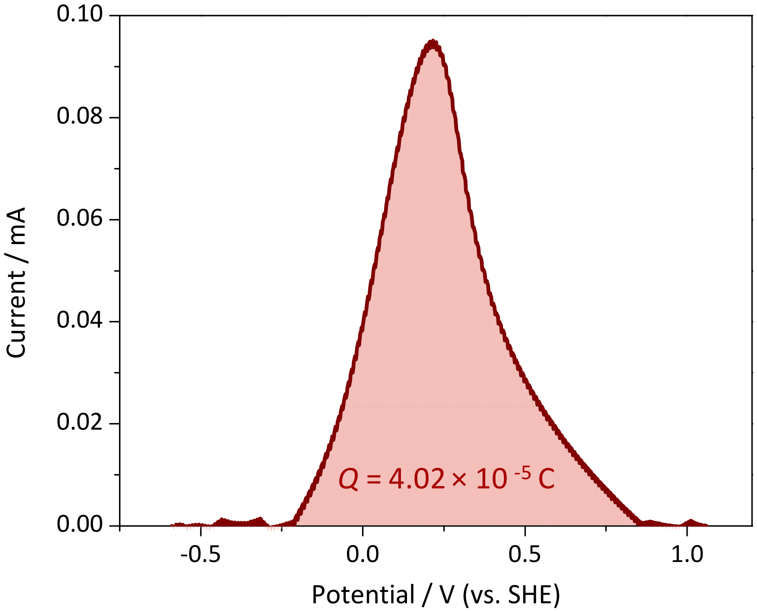

Figure S17. The anodic curve of the first electropolymerization cycle after subtraction of the capacitive current.

The amount of water oxidized per second (mol s^{-1}) was calculated based on the chronoamperogram at 1.21 V. The amount of water oxidized per second (mol s^{-1}) was then normalized by the total number of copper sites on the electrode surface to determine the TOF, the number of water molecules oxidized per Cu^{2+} site per second (s^{-1}).

References

[19] Y. C. Weng, T. H. Cheng, *Zeitschrift fur Naturforsch. - Sect. B J. Chem. Sci.* **2011**, *66*, 279–288.

[20] M. F. Bergamini, D. P. Santos, M. Valnice, B. Zanoni, *J. Electroanal. Chem.* **2013**, *690*, 47–52.

[21] Z. Wang, Y. Chen, W. Dong, J. Zhou, B. Han, J. Jiao, L. Lan, P. Miao, Q. Chen, *Biosens. Bioelectron.* **2018**, *121*, 257–264.

[22] T. M. Ivanova, K. I. Maslakov, A. A. Sidorov, M. A. Kiskin, R. V. Linko, S. V. Savilov, V. V. Lunin, I. L. Eremenko, *J. Electron Spectros. Relat. Phenomena* **2020**, *238*, 146878.

[23] M. C. Biesinger, L. W. M. Lau, A. R. Gerson, R. S. C. Smart, *Appl. Surf. Sci.* **2010**, *257*, 887–898.

[24] D. Zheng, Z. Gao, X. He, F. Zhang, L. Liu, *Appl. Surf. Sci.* **2003**, *211*, 24–30.

[25] Z. H. Yang, X. Lei, G. Jiang, X. Zhang, *RSC Adv.* **2023**, *13*, 15274–15279.

[26] M. A. Ali, A. Hassan, G. C. Sedenho, R. V Gonçalves, D. R. Cardoso, F. N. Crespilho, *J. Phys. Chem. C* **2019**, *123*, 16058−16064.

[27] R. I. R. Blyth, H. Buqa, F. P. Netzer, M. G. Ramsey, J. O. Besenhard, P. Golob, M. Winter, *Appl. Surf. Sci.* **2000**, *167*, 99–106.

[28] A. Torreggiani, M. Tamba, S. Bonora, G. Fini, *Biopolym. - Biospectroscopy Sect.* **2003**, *72*, 290–298.

[29] A. Barth, *Prog. Biophys. Mol. Biol.* **2000**, *74*, 141–173.

[30] E. Prenesti, S. Berto, *J. Inorg. Biochem.* **2002**, *88*, 37–43.

[31] S. R. De Andrade, M. A. Couto, *Spectrochim. Acta Part A* **1999**, *55*, 1185–1191.

[32] K. Hasegawa, T. A. Ono, T. Noguchi, *J. Phys. Chem. A* **2002**, *106*, 3377–3390.

[33] Q. Yang, W. Sui, D. Fan, G. Li, *Adv. Mater. Res.* **2013**, *781–784*, 89–92.

[34] H. A. Younus, Y. Zhang, M. Vandichel, N. Ahmad, K. Laasonen, F. Verpoort, C. Zhang, S. Zhang, *ChemSusChem* **2020**, *13*, 5088–5099.

[35] A. M. Geer, C. Musgrave, C. Webber, R. J. Nielsen, B. A. McKeown, C. Liu, P. P. M. Schleker, P. Jakes, X. Jia, D. A. Dickie, J. Granwehr, S. Zhang, C. W. Machan, W. A. Goddard, T. B. Gunnoe, *ACS Catal.* **2021**, *11*, 7223–7240.

[36] K. J. Fisher, K. L. Materna, B. Q. Mercado, R. H. Crabtree, G. W. Brudvig, *ACS Catal.* **2017**, *7*, 3384–3387.

[37] N. J. Finnerty, F. B. Bolger, *Bioelectrochemistry* **2018**, *119*, 124–135.

[38] L. Rivas, S. Dulay, S. Miserere, L. Pla, S. B. Marin, J. Parra, E. Eixarch, E. Gratacós, M. Illa, M. Mir, J. Samitier, *Biosens. Bioelectron.* **2020**, *153*, 112028.

[39] J. Kong, S. Yu, *Acta Biochim. Biophys. Sin. (Shanghai).* **2007**, *39*, 549–559.

[40] A. Barth, *Biochim. Biophys. Acta* **2007**, *1767*, 1073–1101.
